# Obscured hemorrhagic pancreatitis after orthotopic heart transplantation complicated with acute right heart failure and hepatic dysfunction: a case report

**DOI:** 10.1186/s13019-016-0562-4

**Published:** 2016-12-01

**Authors:** Ting-Wei Lin, Meng-Ta Tsai, Jun-Neng Roan, Yi-Sheng Liu, Hong-Ming Tsai, Chwan-Yau Luo

**Affiliations:** 1 Division of Cardiovascular Surgery, Department of Surgery, National Cheng Kung University Hospital, College of Medicine, National Cheng Kung University, 138 Sheng-Li Road, Tainan City, 70428 Taiwan; 2 Department of Diagnostic Radiology, National Cheng Kung University Hospital, College of Medicine, National Cheng Kung University, Tainan, Taiwan; 3 Cardiovascular Research Center, National Cheng Kung University, Tainan, Taiwan

**Keywords:** Heart transplantation, Pancreatitis, Right heart failure, Case report

## Abstract

**Background:**

Pancreatitis is a serious complication after cardiac surgery and can lead to significant morbidities and mortality. The incidence of pancreatitis is even higher in patients undergoing heart transplantation than in those undergoing other cardiac surgeries. Nevertheless, the clinical presentations of pancreatitis are frequently atypical in these patients.

**Case presentation:**

We report a heart recipient who was complicated with acute right heart failure initially after orthotopic heart transplantation and developed devastating unanticipated hemorrhagic pancreatitis 1 month after the transplantation. This crypto-symptomatic pancreatitis was not diagnosed until massive internal bleeding and hemorrhagic shock occurred, because the typical presentations of acute pancreatitis were masked by the intra-abdominal manifestations caused by right heart failure and congestive liver dysfunction. The patient underwent a successful transarterial embolization.

**Conclusions:**

The causes of pancreatitis after heart transplantation include low cardiac output, immunosuppressant use and cytomegalovirus infection. The typical symptoms of pancreatitis might be not apparent in patients after heart transplantation because of their immunosuppressive status. Furthermore, in patients complicated with right heart failure after transplantation, the manifestation of pancreatitis could be even more obscure. The prompt diagnosis is highly depended on the clinician’s astuteness.

## Background

Pancreatitis is one of the most serious gastrointestinal complications after cardiac surgery that requires a cardiopulmonary bypass, e.g., a heart transplantation [1–3]. Although some risk factors have been identified, the association between post-transplantation acute right heart failure and pancreatitis has rarely been discussed. In such cases, acute pancreatitis might be overlooked because its characteristic symptoms can be concealed by the intra-abdominal presentations caused by right heart failure and subsequent congestive liver dysfunction.

## Case presentation

**Fig. 1:**
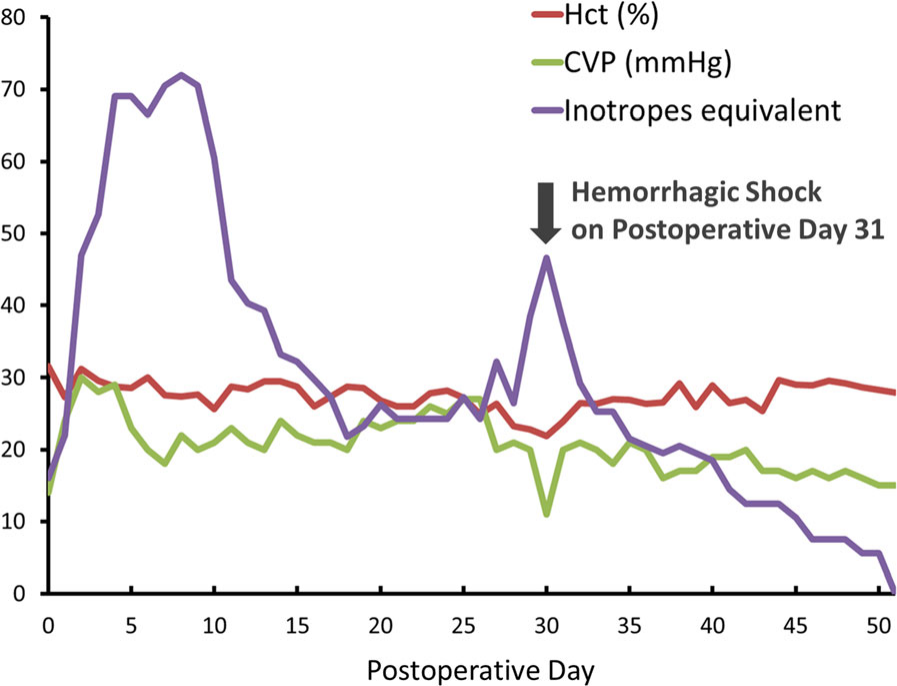
Postoperative series change of inotropes dose, central venous pressure (CVP), and hematocrit (Hct). The initial high dose of inotropes was necessary for acute right heart failure. Shock with decreased CVP and Hct on postoperative day 31 was caused by active retroperitoneal bleeding; the patient was stabilized after an emergency transarterial embolization. The inotropes equivalent was defined as the dose of dopamine + dose of dobutamine + dose of epinephrine × 100 + dose of norepinephrine × 100 (μg/kg/min)

**Fig. 2:**
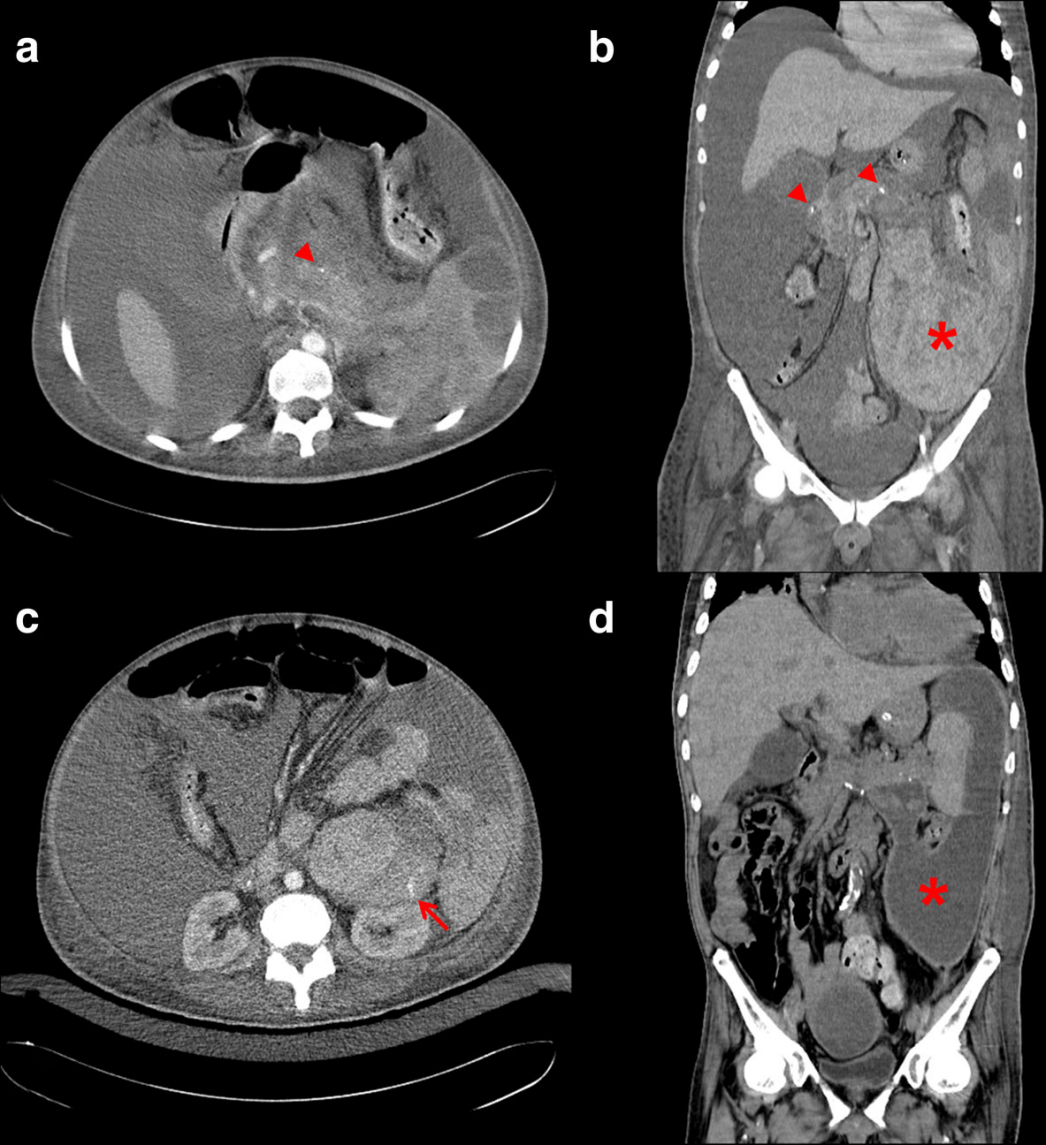
**a** An axial view of the contrast-enhanced abdominal CT shows in the pancreas a heterogeneous pattern and some calcified spots (*arrowhead*) that are compatible with pancreatitis. **b** A coronal view shows intrapancreatic calcifications (*arrowheads*) and one high dense lesion, with a huge hematoma, which occupied the perisplenic and pararenal retroperitoneal space and extended to the lower left abdomen (*asterisk*). **c** A high-resolution image shows one active contrast medium extravasation in the pararenal region (*arrow*). There was also abundant ascites, caused by advanced hepatic failure and right heart failure. **d** A coronal view of contrast-enhanced abdominal CT 2 months later showed fluid accumulated in the space previously occupied by the hematoma (*asterisk*), which is compatible with chronic pancreatitis with one huge and extended pseudocyst, because the fluid had an extremely high concentration of pancreatic enzymes

A 43-year-old man with non-ischemic dilated cardiomyopathy-related end-stage heart failure underwent orthotopic heart transplantation. Before surgery, he has a fluctuated heart function which was dependent on mid-level dose of intravenous inotropes (~5 μg/kg/min dobutamine) and his Interagency Registry for Mechanically Assisted Circulatory Support (INTERMACS) profile was level 3. His preoperative serum creatinine level was 2.3 mg/dL and estimated glomerular filtration rate (eGFR) was 33 ml/min/1.73 m^2^. His serum total bilirubin had risen to 3.2 mg/dL; and his Child-Turcotte-Pugh and Model for End-Stage Liver Disease (MELD) scores were 8 and 23 points, respectively. A right heart hemodynamic study before transplantation revealed that the transpulmonary pressure gradient (TPG) was 11 mmHg and the pulmonary vascular resistance (PVR) was 3.63 Wood units. Moreover, the body weight of the male donor was only 80% of the patient’s. Right heart failure developed on postoperative day 2 and the patient had an extremely high central venous pressure (CVP) level (27 mmHg), abundant ascites, and acute kidney injury that required continuous renal replacement therapy. A fulminant hepatic failure also developed and his serum total bilirubin peaked at 23 mg/dL in the first weeks after his heart transplantation. Abdominal echography excluded biliary tract obstruction and cholelithiasis. Plasmapheresis was done three times because of the patient’s progressive hyperbilirubinemia. Four weeks after the transplantation, his condition stabilized; nevertheless, on postoperative day 31, the patient manifested painful diffuse abdominal distention associated with hypovolemic shock and a decrease in blood hematocrit level (Fig. 1). Contrast-enhanced abdominal computed tomography (CT) showed a heterogeneous pattern of the pancreas and one huge retroperitoneal hematoma with active bleeding in the left pararenal region (Fig. 2a, b and c). Although the serum amylase and lipase levels were both within the normal range at that time, a prior acute pancreatitis episode with subsequent enzymatic destruction of adjacent vasculatures, i.e., hemorrhagic pancreatitis, was suspected. An emergency transarterial embolization (TAE) was done for the actively bleeding left anteroinferior subsegmental renal artery with several pledgets of gelatin sponge (Gelfoam, Pfizer, New York, N.Y.). After bleeding ceased, a conservative treatment strategy for pancreatitis was adopted for this patient since no other complication occurred. Two months later, a second abdominal CT showed calcification of and adjacent fluid accumulation in the pancreas (Fig. 2d), and external drainage of the fluid revealed high amylase and lipase levels (1037 U/L and 4035 U/L, respectively), both of which were compatible with chronic pancreatitis with a pseudocyst. An external drain was kept in situ for an additional 2 months. The patient was discharged from the hospital 6 months after his heart transplantation; at that time, the patient’s right heart function had improved: CVP was ca. 6 mmHg, eGFR was ca. 60 ml/min/1.73 m^2^, and he had no more hyperbilirubinemia.

## Conclusions

The incidence of pancreatitis in patients who have undergone orthotopic heart transplantation ranges from 2 to 18%, which is 15 to 25 times higher than after other cardiac procedures and causes high mortality [1–3] Nevertheless, a diagnosis of pancreatitis after heart transplantation might be difficult and often delayed because of its obscured clinical presentation [1]. Immunosuppressive status after transplantation conceals the systemic inflammatory response and contributes to so-called crypto-symptomatic pancreatitis [3]. Some immunosuppressants, such as corticosteroid, azathioprine, cyclosporine, and (rarely) tacrolimus, are known to be associated with pancreatitis [2, 3]; however, drug-related pancreatitis was not likely in this patient because he eventually recovered without stopping any of these medications. Cytomegalovirus infection is proposed to be another important etiology of pancreatitis in patients who have undergone a transplantation [2, 3], but this was excluded by the patient’s negative antigenemia. Low cardiac output after cardiac surgery is known to be a risk factor for developing postoperative pancreatic cell injury [4]. Decompensated liver failure is also frequently associated with acute pancreatitis: the incidence in more than 30% [5]. Hence, pancreatitis in patients with acute right heart failure is reasonably contributed by a right-side pumping failure-related low cardiac output and congestive hepatic dysfunction. However, in patients who develop right heart failure after a heart transplantation, clinical recognition of pancreatitis could be even more difficult, not only because of the suppressed inflammatory response, but also because the typical presentations of acute pancreatitis are masked by the intra-abdominal manifestations caused by right heart failure and hepatic dysfunction, such as abundant ascites, impaired digestion, and abdominal tenderness, as in our patient. In the presented case, the diagnosis of pancreatitis was not established until massive internal bleeding and hemorrhagic shock occurred. A high index of suspicion, regular monitoring of serum pancreatic enzymes, and more liberal use of abdominal CT or magnetic resonance imaging are recommended to identify this undesired complication for patients who develop right heart failure after a heart transplantation. Finally, utility of mechanical circulatory supports, i.e., extracorporeal membrane oxygenation (ECMO) or ventricular assist devices (VADs), should be advocated in the early postoperative phase in the presented case developing considerable postoperative right heart failure, to avoid subsequent multiorgan dysfunction and the devastating hemorrhagic pancreatitis.

## Abbreviations

CT: Computed tomography
CVP: Central venous pressure
ECMO: Extracorporeal membrane oxygenation
eGFR: Estimated glomerular filtration rate
INTERMACS: Interagency Registry for Mechanically Assisted Circulatory Support
MELD: Model for End-Stage Liver Disease
PVR: Pulmonary vascular resistance
TAE: Transarterial embolization
TPG: Transpulmonary pressure gradient
VAD: Ventricular assist device
